# Activation of RMTg projections to the VTA reverse cocaine-induced molecular adaptation in the reward system

**DOI:** 10.1038/s41398-024-02763-9

**Published:** 2024-01-19

**Authors:** A. Khayat, R. Yaka

**Affiliations:** Institute for Drug Research (IDR), School of Pharmacy, Faculty of Medicine, The Hebrew University of Jerusalem, Jerusalem, 91120 Israel

**Keywords:** Addiction, Molecular neuroscience

## Abstract

The rostromedial tegmental nucleus (RMTg) plays a crucial role in regulating reward-related behavior by exerting inhibitory control over the ventral tegmental area (VTA). This modulation of dopamine neuron activity within the VTA is essential for maintaining homeostasis in the reward system. Recently we have shown that activation of RMTg projections to the VTA during the acquisition of cocaine-conditioned place preference (CPP) reduces the rewarding properties of cocaine and decreases VTA dopamine neuron activity. By inhibiting dopamine neurons in the VTA, we hypothesized that RMTg projections hold the potential to restore reward system homeostasis disrupted by repeated cocaine use, and attenuate molecular adaptations in the reward system, including alterations in signaling pathways. Our study demonstrates that enhancing the GABAergic inputs from the RMTg to the VTA can mitigate cocaine-induced molecular changes in key regions, namely the VTA, nucleus accumbens (NAc), and prefrontal cortex (PFC). Specifically, we found that cocaine-induced alteration in the phosphorylation state of ERK (pERK) and GluA1 on serine 845 (S845) and serine 831 (S831), that play a major role in plasticity by controlling the activity and trafficking of AMPA receptors, were significantly reversed following optic stimulation of RMTg afferents to the VTA. These findings highlight the therapeutic potential of targeting the RMTg-VTA circuitry for mitigating cocaine reward. Ultimately, this research may pave the way for novel therapeutic interventions that restore balance in the reward system and alleviate the detrimental effects of cocaine.

## Introduction

Drug addiction is a persistent brain condition characterized by uncontrollable urges to seek and use drugs, even in the face of harmful outcomes. This debilitating issue leads to significant societal, financial, and health consequences due to the inability to regulate drug consumption [1]. Although extensive research has been devoted to understanding the neurochemical processes involved in addiction, effective treatment solutions remain elusive. The brain’s reward system, comprising the VTA, NAc, and PFC [2], plays a central role in reinforcing rewarding behaviors and regulating punishment [3]. The VTA is the main source of dopamine in the reward system and is crucial for motivation, reward, and addiction [4, 5]. Therefore, its modulation is of great importance. It receives excitatory and inhibitory inputs from various brain regions. For example, the VTA receives excitatory inputs from the PFC [6, 7], laterodorsal tegmental nucleus [8, 9], lateral habenula (LHb) [7, 10], and bed nucleus of the stria terminalis [11], which enhance the firing rate of dopamine (DA) neurons. In addition, it receives GABAergic inhibitory inputs from several sources, including the RMTg [12–14], the NAc [15, 16], the ventral pallidum [17], and GABAergic interneurons within the VTA itself [18–20]. GABAergic synapses are actually the primary type of synapse in midbrain dopamine neurons, highlighting the crucial role of GABAergic transmission in regulating dopamine neuronal activity [4, 21–24].

In our study, we focused on the GABAergic inputs from the RMTg, which is also referred to as the “tail of the VTA. This region, located near the VTA in the midbrain, consists mainly of GABAergic neurons. The RMTg has gained recognition for its critical role in aversive behaviors [12], particularly those associated with behavioral inhibition, such as freezing and avoidance. The functional significance of the RMTg lies in its ability to modulate DA signaling and influence processes related to reward. By exerting inhibitory control over the VTA [13, 14, 16, 17], the RMTg can regulate the activity of DA neurons and the release of DA in target regions such as the NAc and PFC [18]. Importantly, the RMTg receives substantial input from the lateral habenula (LHb), enabling it to translate glutamatergic signals from the LHb into GABAergic inhibition of VTA dopamine neurons [25]. This connectivity allows the RMTg to participate in the regulation of reward, motivation, aversion, reward-omission, and behavioral avoidance [13, 14, 26].

Drugs of abuse, like cocaine, induce neuroadaptations in the reward system, leading to addiction. These neuroadaptations primarily occur in the mesocorticolimbic DA system, resulting in increased sensitivity to drug-related cues, impulsive decision-making, and habit-like behaviors [27, 28]. The underlying molecular changes involve synaptic plasticity, modifications in glutamatergic signaling [29–31], and dysregulation of protein kinase activity, such as ERK [32–36]. Understanding these molecular adaptations within the reward system is crucial for the development of effective interventions for drug reward and addiction.

In our previous research, we presented compelling evidence demonstrating that activating the inhibitory inputs from the RMTg to the VTA has a significant impact on reducing cocaine- CPP and decreasing neuronal activity in VTA DA neurons [37]. Building upon these findings, our current study aimed to delve deeper into the underlying mechanisms involved. We sought to investigate both the behavioral and molecular consequences of activating the projections from the RMTg to the VTA in the context of cocaine reward. Specifically, we focused our investigation on three reward-related regions: the VTA, NAc, and PFC. To achieve our research objectives, we employed two key methodologies: optogenetics and the repeated-exposure place preference (RePP) paradigm. Optogenetics enabled us to selectively activate the specific projections originating from the RMTg and targeting the VTA, thereby allowing us to examine the effects within the reward-related regions of interest. Additionally, we utilized a modified version of the traditional CPP paradigm, known as the RePP paradigm. This paradigm involved subjecting the experimental subjects to repeated exposures within the drug-paired environment. By utilizing the RePP paradigm, we aimed to achieve a more efficient assessment of the behavioral effects resulting from the activation of the RMTg-VTA projections in the context of cocaine-induced neuroadaptations.

In summary, our study seeks to expand our understanding of the underlying mechanisms involved in drug reward by investigating the consequences of activating the RMTg-VTA projections. Through the utilization of optogenetics and the RePP paradigm, we hope to shed light on the behavioral and molecular implications of this activation, particularly within the VTA, NAc, and PFC.

## Materials and methods

### Animals

Sprague Dawley rats (Harlen laboratories) weighing between 50–90 g were group-housed in the animal facility under specific conditions. The ambient temperature was maintained at 22 °C, and the rats were kept on a 12-h light/dark cycle, with the lights turned on at 7:00 a.m. The animals had access to food and water ad libitum, allowing them to consume as much as they desired. During the behavioral studies, the experiments were conducted in a dark room with only red light present. This choice of light is undetectable by rodents. All procedures performed in this study were approved by the Institutional Animal Care Committee (IACUC) of the Hebrew University in Jerusalem, Israel. Furthermore, the experimental design and execution were conducted in a manner that prioritized the welfare and minimized any potential discomfort experienced by the animals. Rats were excluded from analysis if weight loss of greater than 10% occurred and randomization was carried out where feasible (via Excel). The same investigator analyzed the data.

### Stereotactic surgery

On the day of surgery, the rats were anesthetized using a combination of xylazine and ketamine (in a ratio of 0.15:0.85). A microinjection needle with a diameter of 33 gauge was connected to a 10 μl syringe (Hamilton, USA). The needle was inserted unilaterally into the RMTg (coordinates: AP: −6.3, ML: 0.8, DV: −7.6). A purified adeno-associated virus (AAV) (2 × 10^9^ units; 1 μl vol) encoding hChR2(H134R)-m-Cherry under the control of the human synapsin 1 promoter was injected for a duration of 5 min, with the syringe left in place for an additional 7 min. The injections were performed using a Digital Lab Standard™ Stereotactic instrument (Stoelting Co. IL, USA). Following the surgery, the rats were housed individually in separate cages for a period of 2 weeks to allow for recovery and complete expression of the viral vector. Two weeks after the injection of the channel rhodopsin-containing virus into the RMTg the animals underwent another surgery in which an optic fiber was implanted above the VTA (coordinates: AP: −5.1, ML: 0.7, DV: −8.1). The rats were given 1 week to recover before the initiation of the behavioral experiments.

### Conditioning apparatus

The CPP apparatus used in this study (Med Associates, USA) consists of a three-compartment elongated rectangular apparatus. The middle compartment serves as a neutral zone, measuring 12 cm × 21 cm, and it separates the two elongated compartments. One of the compartments is characterized by white-colored walls and wire mesh flooring measuring 28 cm × 21 cm. The other compartment features black-colored walls and steel rod flooring of the same dimensions. To track the time spent in each compartment, Infrared photobeam crossings were positioned at the bottom of the walls. These photobeams detect the movement of the animals and record the time spent in each compartment during the CPP experiments.

### Repeated-exposure place preference (RePP)

Three weeks post-injection of the channel rhodopsin-containing virus to the RMTg, rats underwent a habituation session in the CPP apparatus. The least preferred compartment for each subject was then designated as the drug-paired compartment (biased). Following the habituation session, rats underwent six conditioning sessions over three consecutive days, with two sessions per day. They were randomly assigned to one of four groups: (1) Saline control group: These rats received saline (1 ml/kg, i.p.) in both sessions without any optic stimulation. (2) Saline control group with optic stimulation: These rats received saline (1 ml/kg, i.p.) in both sessions, with optic stimulation only during the morning sessions. (3) Cocaine control group: These rats received cocaine (15 mg/kg, i.p.) in the morning session and saline (1 ml/kg, i.p.) in the afternoon session, without any optic stimulation. (4) Test group: These rats received cocaine (15 mg/kg, i.p.) in the morning session and saline (1 ml/kg, i.p.) in the afternoon session, with optic stimulation only during the cocaine sessions. In all cases, an optic cannula was connected to the light source, and optic stimulation was administered using an Ultra High-Power LED (Prizmatix, Israel). For RMTg stimulation, the optic stimulation protocol involved delivering stimulation at 50 Hz for half a second every second [38]. On the fifth day of the experiment, a CPP test was conducted to assess the CPP score. The CPP score was calculated as a percentage, using the following formula: CPP score = 100 * [(time spent in the drug/saline-paired compartment) – (time spent in the saline-paired compartment)]/[(time spent in the drug/saline-paired compartment) + (time spent in the saline-paired compartment)]. This CPP score measurement provided an assessment of the CPP, indicating the relative preference for the drug-paired compartment compared to the saline-paired compartment.

### Western blot analysis

Twenty minutes following the CPP test, rats were anesthetized using isoflurane (2–3 min). Following anesthesia, they were decapitated, and their brains were immediately removed. Coronal sections, each approximately 1 mm thick, containing the VTA, NAc, and PFC were obtained. These sections were then microdissected bilaterally on an ice-cold platform and promptly transferred to liquid nitrogen to preserve the tissue. The microdissected tissue samples were homogenized using a homogenization buffer consisting of 320 mM sucrose, 10 mM Tris-HCl (pH 7.4), 1 mM EDTA, 1 mM EGTA, a protease inhibitor cocktail (Sigma, P8340), and phosphatase inhibitors (1 mM Na_3_VO_4_ and 5 mM NaF by Sigma-Merck, Darmstadt, Germany). The purpose of the homogenization step is to break down the tissue and release the proteins of interest. To determine the total protein concentration in the brain homogenates, the Pierce™ BCA Protein Assay Kit (Pierce, IL, USA) was utilized, with bovine serum albumin (BSA) as a standard. The protein samples were then boiled for 5 min at 95 °C to denature the proteins and loaded (20 µg protein/lane) onto 8-12.5% SDS-PAGE gels. Electrophoresis was performed to separate the proteins based on their molecular weight, and the separated proteins were transferred onto a nitrocellulose blotting membrane (TAMAR, Germany). The purpose of this transfer is to immobilize the proteins on the membrane for subsequent antibody detection. Following the transfer, the membranes were incubated at room temperature for 1 h in a blocking buffer containing 5% nonfat dry milk. This step helps prevent nonspecific binding of antibodies. The membranes were then incubated overnight at 4 °C with the desired primary antibodies. The primary antibodies used in this study were Phospho ERK (Thr202/Tyr204) (cat. no. 91015; 1:1,500; Cell Signaling), ERK (cat. no. 9102 s; 1:1000; Cell Signaling), GluR1 (cat. no. AB1504; 1:2,000; Merck), Anti-phospho-GluR1 (Ser845) (cat. no. AB 5849; 1:500; Merck), Anti-phospho-GluR1 (Ser831) (cat. no. 04-823; 1:500; Merck), and Beta Actin (cat. no. ab8227; 1:7,000; Abcam). After primary antibody incubation, the membranes were washed to remove unbound antibodies and then incubated with the appropriate HRP-conjugated secondary antibody for 1.5 h at room temperature. The secondary antibody recognizes and binds to the primary antibody, allowing for the detection of the target proteins. The blots on the membrane were detected and quantified using the Clarity™ western ECL system and the BioRad ChemiDoc™ XRS+ Imaging system (BioRad, CA, USA). This detection method utilizes chemiluminescence to visualize the bound antibodies on the membrane and quantify the protein bands.

### Immunohistochemistry

Rats were deeply anesthetized with isoflurane and then transcardially perfused with phosphate-buffered saline (PBS) followed by 4% paraformaldehyde (Sigma, Germany) in PBS. The brains were carefully extracted and immersed in 4% paraformaldehyde overnight and transferred to a solution of 30% sucrose in ddH20. Horizontal VTA-containing brain slices with a thickness of thirty micrometers were obtained using a cryostat (Leica). The brain sections were immediately permeabilized and blocked using a solution of 0.3% Triton and 5% Normal Donkey Serum (NDS) in PBS for a duration of 1 h. To stain dopaminergic cells in the VTA, the Tyrosine Hydroxylase primary antibody (TH 1:400, MAB318, Millipore) was directly added to the blocking solution and allowed to incubate overnight at 4 °C. The next day, sections were washed three times in PBS containing 0.3% Triton and 1% NDS. Sections were then transferred to a washing solution containing the appropriate secondary antibody (1:400, Alexa flour 488; 715-485-151, Jackson Immunoresearch) and incubated at room temperature for 2 h. After the incubation, the sections were washed three times again using the washing solution. Finally, the sections were mounted on Superfrost slides using IMMU MOUNT (Thermo Scientific, USA). The acquired images were captured using a Fluoview FV10i confocal microscope with either a 10× or 60× objective. The images were then analyzed using Olympus Fluoviewer software.

### Statistical analysis

For the behavioral and biochemical analysis, the effect of treatment was assessed using a one-tailed *t*-test, and a two-tailed Student’s unpaired *t*-test for pairwise comparisons. These tests were chosen based on the specific hypotheses and research questions being investigated. To analyze multiple comparisons between groups, a two-way analysis of variance (ANOVA) was employed. In cases where significant differences were observed in the two-way ANOVA, post hoc Tukey honestly significant difference (HSD) tests were conducted. This approach allowed us to thoroughly examine the impact of the treatment and identify any significant variations between the experimental groups. Data are presented as mean ± SEM (standard error of the mean). The accepted level of significance for all tests was set at *P* < 0.05, which is denoted by asterisks in the figures. The figure legends provide information regarding the group sizes and significant differences observed. The statistical analysis was performed using Prism 8 software.

## Results

### Optic stimulation of RMTg afferents to the VTA during the acquisition phase of RePP significantly reduced cocaine CPP

**Fig. 1:**
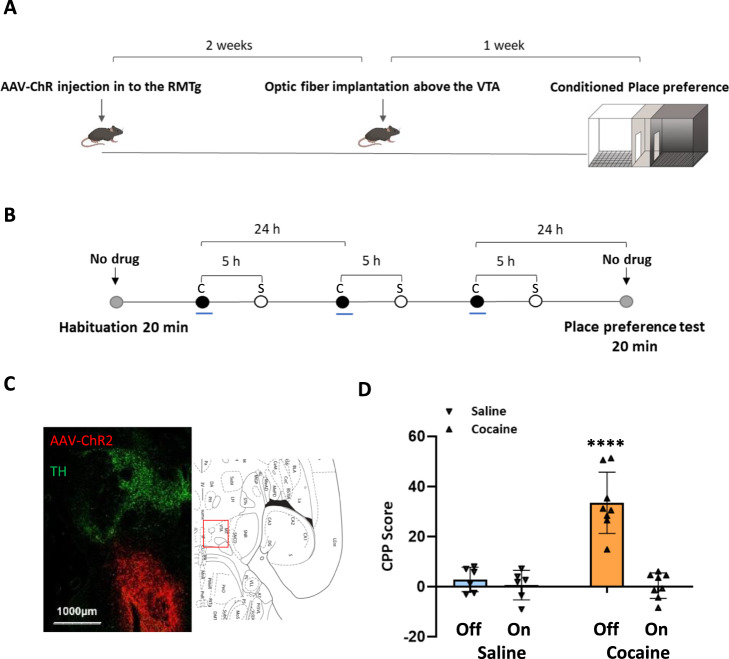
Activation of RMTg afferents during cocaine exposure reduced the expression of CPP. **A** An illustration demonstrating AAV-ChR2 injection in the RMTg and optic fiber implantation above the VTA. This setup allows for the optic stimulation of VTA-innervating afferents during RePP. **B** For the CPP study, rats were randomly assigned to four groups. Following habituation (Hab.), the first control group received an injection of saline in both compartments (1 ml/kg) without optic stimulation. The second control group received an injection of saline in both compartments (1 ml/kg) and underwent optic stimulation. The third control group received an injection of saline vs cocaine (15 mg/kg) 5 h apart without optic stimulation. Finally, the fourth group received an injection of saline vs cocaine (15 mg/kg) 5 h apart and underwent optic stimulation during conditioning. One day following the termination of conditioning (day 5, test), the rats were tested for their preference for the cocaine-paired compartment. ( = Rats confined to black or white compartment after injection of saline (1 ml/kg) or cocaine (15 mg/kg) with or without optic stimulation,  = Rats confined to black or white compartment after injection of saline (1 ml/kg),  = Free exploration). **C** VTA-containing horizontal brain slices were stained with tyrosine hydroxylase (TH, DA neurons marker, green) and m-cherry (a marker of neurons expressing AAV-ChR2, red) taken from rats 45 min following the test. **D** Graph depicting preference values, expressed as the mean CPP score ± SEM, in RMTg-injected animals (*n* = 6, 6, 8, and 8 rats for the saline–optic stimulation (Off), saline+optic stimulation (On), cocaine–optic stimulation (Off), and cocaine+optic stimulation (On) groups, respectively) on the test day.****Cocaine–optic stimulation group differs significantly from all the other groups (*P* < 0.0001). Tukey’s multiple comparison test was used for post hoc analysis. (Off refers to –optic stimulation, whereas On refers to +optic stimulation).

In a previous study, we demonstrated that exogenously activating RMTg afferents in the VTA during the acquisition phase of cocaine-conditioned reward resulted in the elimination of animals’ preference for the cocaine-associated chamber, as well as a reduction in neuronal activity in the VTA [37]. Building upon these findings, our current study aimed to further investigate the effects of RMTg activation on cocaine-induced molecular changes in reward-related brain areas, specifically the VTA, NAc, and PFC. To examine these effects, we conducted a shortened CPP experiment using four distinct groups of animals. After a period of 45 min following the RePP test, the animals were sacrificed, and the desired areas (VTA, NAc, and PFC) were collected for Western blot analysis. Consistent with the previous longer CPP experiment, the RePP test yielded similar results. Animals that received cocaine without optic stimulation displayed a significant preference for the cocaine-associated chamber (one-sample *t*-test: *t* = 7.749, *P* = 0.0001). However, when RMTg afferents were stimulated during cocaine conditioning, their preference for the cocaine-associated chamber was abolished (one-sample *t*-test: *t* = 0.2872, *P* > 0.05). Notably, optic stimulation of the RMTg in saline-injected animals did not have any impact on their preference (unpaired *t*-test: *t* = 0.7256, *P* > 0.05). Post hoc Tukey HSD tests confirmed that the group receiving cocaine without optic stimulation showed significant differences compared to the other groups (*P* < 0.0001), this conclusion was based on the results of a two-way ANOVA test [*F*_OPTIC STIMULATION_ (1,24) = 33.68, *P* < 0.0001, *F*_GROUP_(1,24) = 25.07, *P* < 0.0001, *F*_INTERACTION_ (1,24) = 25.60, *P* < 0.0001] (Fig. 1C, D).

### Enhanced GABAergic tone from the RMTg during conditioning to cocaine reverses cocaine-induced molecular changes in the VTA, NAc, and PFC

We next investigated the expression and function of key proteins involved in plasticity following optic stimulation of RMTg-VTA projections. Specifically, we focused on examining the alteration in the expression and function of ERK (extracellular signal-regulated kinase) and the GluA1 subunit of AMPA receptor. ERK is a signaling protein that plays a crucial role in various cellular processes, including neuronal plasticity. Its phosphorylation state (activation) is often assessed to determine its involvement in signaling pathways indicating its activation and subsequent involvement in cellular events [35, 38]. The GluA1 subunit of the AMPA receptor is a key component involved in mediating excitatory neurotransmission in the brain. Phosphorylation of specific serine residues on the intracellular tail of GluA1, such as S831 and S845, is known to be critically involved in the trafficking and function of GluA1 and can influence its localization to the synapse and its overall synaptic efficacy [28, 39, 40].

**Fig. 2:**
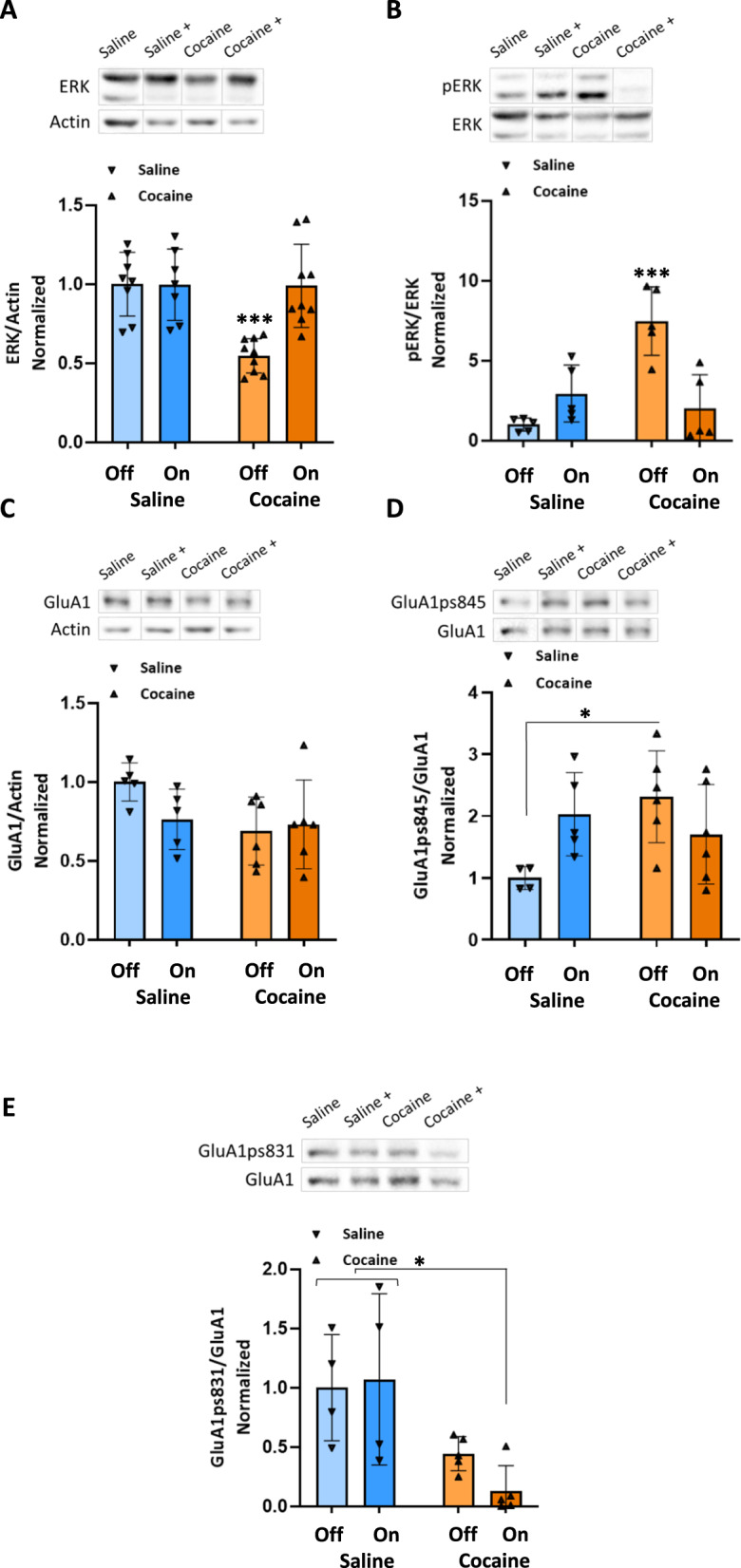
Enhanced GABAergic tone from the RMTg during conditioning to cocaine attenuates changes in ERK, PERK, and GluA1ps845 in the VTA. All rats were euthanized 45 min after the RePP test. Then, their VTA was taken out and homogenized. Samples were resolved by SDS-PAGE, and WB membranes were probed with the appropriate antibodies. **A** The bar histograms depict the level of ERK divided by Actin, rats treated with cocaine (15 mg/kg) displayed significantly lower values compared to all other groups (*P* < 0.001), as determined by a two-way ANOVA test [*F*_OPTIC STIMULATION_ (1,29) = 9.199, P < 0.01, *F*_GROUP_(1,29) = 10.13, *P* < 0.01, *F*_INTERACTION_ (1,29) = 9.441, P < 0.01, followed by Tukey’s multiple comparison test]. (*n* = 8, 7, 9, and 9 for saline -, saline +, cocaine-, and cocaine+ groups, respectively). **B** The bar histograms depict the level of pERK (Thr202/Tyr204) divided by ERK protein, rats treated with cocaine (15 mg/kg) displayed significantly higher values compared to all other groups (*P* < 0.001), as determined by a two-way ANOVA test [*F*_OPTIC STIMULATION_ (1,16) = 5.075, *P* < 0.05, *F*_GROUP_(1,16) = 12.3, *P* < 0.01, *F*_INTERACTION_ (1,16) = 22.18, P < 0.001, followed by Tukey’s multiple comparison test]. (*n* = 5 for all groups). **C** The bar histograms depict the level of GluA1 divided by Actin. No significant difference was found [F_OPTIC STIMULATION_ (1,18) = 1.112, *P* > 0.05, *F*_GROUP_(1,18) = 3.486, *P* > 0.05, *F*_INTERACTION_ (1,18) = 2.292, *P* > 0.05, two-way ANOVA followed by Tukey’s multiple comparison test]. (*n* = 5 for the saline groups, *n* = 6 for the cocaine groups). **D** The bar histograms depict the level of GluA1ps845 divided by GluA1 protein, rats treated with cocaine (15 mg/kg) displayed significantly higher values compared to saline-treated rats (*P* < 0.05), as determined by a two-way ANOVA test [*F*_OPTIC STIMULATION_ (1,17) = 0.5023, *P* > 0.05, *F*_GROUP_(1,17) = 2.699, P > 0.05, *F*_INTERACTION_ (1,17) = 7.414, *P* < 0.05, followed by Tukey’s multiple comparison test]. (*n* = 4, 5, 6, and 6 for saline–, saline +, cocaine-, and cocaine+ groups, respectively). **E** The bar histograms depict the level of GluA1ps831 divided by GluA1 protein, rats treated with cocaine (15 mg/kg) with optic stimulation displayed significantly lower values compared to the saline with optic stimulation and saline without optic stimulation groups (*P* < 0.05), as determined by a two-way ANOVA test. A nonsignificant reduction was found in the cocaine-treated group compared to the saline with optic stimulation and saline without optic stimulation groups [*F*_OPTIC STIMULATION_ (1,14) = 0.3760, *P* > 0.05, *F*_GROUP_(1,14) = 14.33, *P* < 0.01, *F*_INTERACTION_ (1,14) = 0.9373, *P* > 0.05, followed by Tukey’s multiple comparison test]. (*n* = 4 for the saline groups, *n* = 5 for the cocaine groups). (Off refers to - optic stimulation, whereas +On refers to +optic stimulation).

As shown in Fig. 2, in the VTA, rats treated with cocaine displayed significantly higher values of pERK compared to all other groups (*P* < 0.001), as determined by a two-way ANOVA test [*F*_OPTIC STIMULATION_ (1,16) = 5.075, *P* < 0.05, *F*_GROUP_(1,16) = 12.3, *P* < 0.01, *F*_INTERACTION_ (1,16) = 22.18, *P* < 0.001], followed by Tukey’s multiple comparison test. Additionally, these rats displayed a significant increase in GluA1ps845 compared to the saline-treated rats (*P* < 0.05), as determined by a two-way ANOVA test [*F*_OPTIC STIMULATION_ (1,17) = 0.5023, *P* > 0.05, *F*_GROUP_(1,17) = 2.699, *P* > 0.05, *F*_INTERACTION_ (1,17) = 7.414, *P* < 0.05]. Furthermore, enhancing the GABAergic tone originating from the RMTg during cocaine conditioning attenuates changes in ERK and pERK in the VTA (Fig. 2A, B). This enhancement also significantly decreased GluA1 phosphorylation at S831 compared to the saline with optic stimulation and saline without optic stimulation groups (*P* < 0.05), as determined by a two-way ANOVA test [*F*_OPTIC STIMULATION_ (1,14) = 0.3760, *P* > 0.05, *F*_GROUP_(1,14) = 14.33, *P* < 0.01, *F*_INTERACTION_ (1,14) = 0.9373, *P* > 0.05], followed by Tukey’s multiple comparison test.

**Fig. 3:**
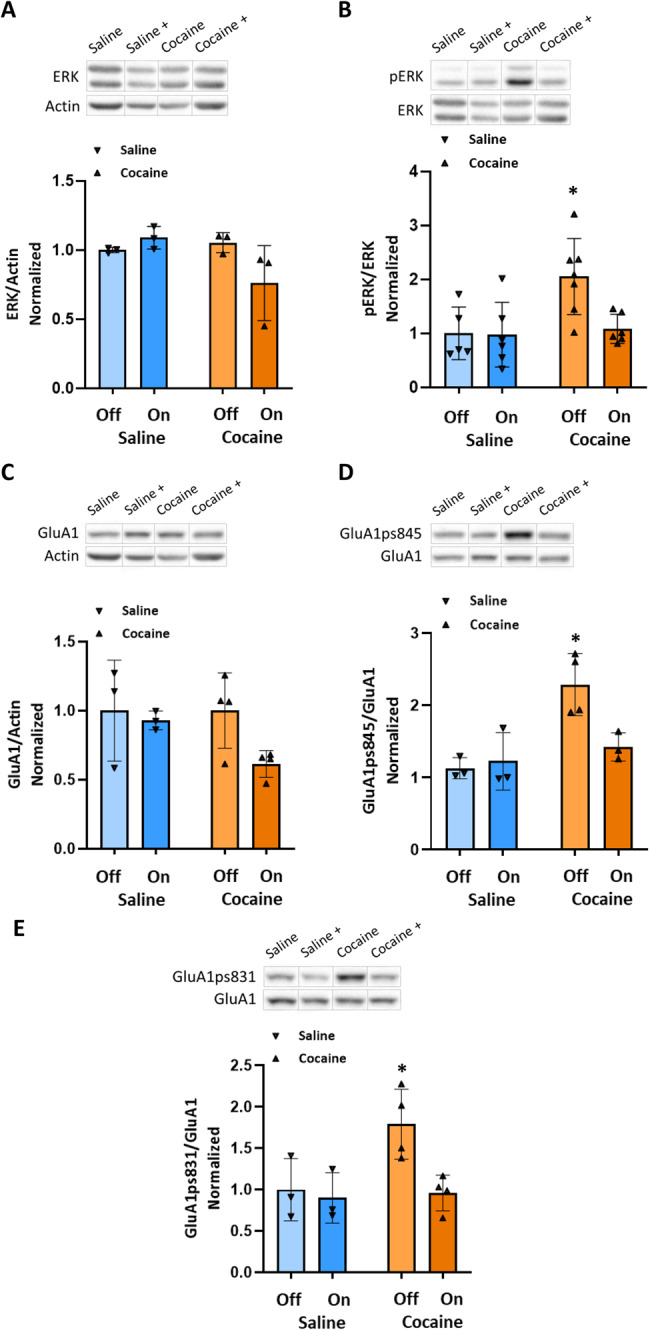
Enhanced GABAergic tone from the RMTg during conditioning to cocaine attenuates changes in PERK, GluA1ps845, and GluA1ps831 in the NAc. All rats were euthanized 45 min after the RePP test. Then, their NAc was taken out and homogenized. Samples were resolved by SDS-PAGE, and WB membranes were probed with the appropriate antibodies. **A** The bar histograms depict the level of ERK divided by Actin, no significant difference was found (*P* > 0.05), as determined by a two-way ANOVA test [*F*_OPTIC STIMULATION_ (1,8) = 1.456, *P* > 0.05, *F*_GROUP_ (1,8) = 2.635, *P* > 0.05, *F*_INTERACTION_ (1,8) = 5.095, *P* > 0.05, followed by Tukey’s multiple comparison test]. (*n* = 3 for all groups). **B** The bar histograms depict the level of pERK (Thr202/Tyr204) divided by ERK protein, rats treated with cocaine (15 mg/kg) displayed significantly higher values compared to all other groups (*P* < 0.05), as determined by a two-way ANOVA test [*F*_OPTIC STIMULATION_ (1,20) = 4.809, *P* < 0.05, *F*_GROUP_ (1,20) = 6.547, *P* < 0.05, *F*_INTERACTION_ (1,20) = 4.335, *P* = 0.0504, followed by Tukey’s multiple comparison test]. (*n* = 5, 6, 7, and 6 for saline–, saline +, cocaine–, and cocaine+ groups, respectively). **C** The bar histograms depict the level of GluA1 divided by Actin, no significant difference was found [*F*_OPTIC STIMULATION_ (1,10) = 3.39, *P* > 0.05, *F*_GROUP_ (1,10) = 1.603, *P* > 0.05, *F*_INTERACTION_ (1,10) = 1.605, *P* > 0.05, two-way ANOVA followed by Tukey’s multiple comparison test]. (*n* = 3 for saline groups, *n* = 4 for cocaine groups). **D** The bar histograms depict the level of GluA1ps845 divided by GluA1 protein, rats treated with cocaine (15 mg/kg) displayed significantly higher values compared to all other groups (*P* < 0.05), as determined by a two-way ANOVA test [*F*_OPTIC STIMULATION_ (1,9) = 4.275, *P* > 0.05, *F*_GROUP_ (1,9) = 13.35, *P* < 0.01, *F*_INTERACTION_ (1,9) = 6.738, *P* < 0.05, followed by Tukey’s multiple comparison test]. (*n* = 3, 3, 4, and 3 for saline–, saline+, cocaine–, and cocaine+ groups, respectively). **E** The bar histograms depict the level of GluA1ps831 divided by GluA1 protein, rats treated with cocaine (15 mg/kg) without optic stimulation displayed significantly higher values compared to all other groups (*P* < 0.05), as determined by a two-way ANOVA test. [*F*_OPTIC STIMULATION_ (1,10) = 6.500, *P* < 0.05, *F*_GROUP_ (1,10) = 5.396, *P* < 0.05, *F*_INTERACTION_ (1,10) = 4.019, *P* > 0.05, followed by Tukey’s multiple comparison test]. (*n* = 3 for the saline groups, *n* = 4 for the cocaine groups). (Off refers to –optic stimulation, whereas On refers to +optic stimulation).

In the NAc (Fig. 3), rats treated with cocaine displayed significantly higher values of pERK compared to all other groups (*P* < 0.05), as determined by a two-way ANOVA test [*F*_OPTIC STIMULATION_ (1,20) = 4.809, *P* < 0.05, *F*_GROUP_ (1,20) = 6.547, *P* < 0.05, *F*_INTERACTION_ (1,20) = 4.335, *P* = 0.0504], followed by Tukey’s multiple comparison test. Additionally, these rats displayed significantly higher values of GluA1ps845 compared to all other groups (*P* < 0.05), as determined by a two-way ANOVA test [*F*_OPTIC STIMULATION_ (1,9) = 4.275, *P* > 0.05, *F*_GROUP_ (1,9) = 13.35, *P* < 0.01, *F*_INTERACTION_ (1,9) = 6.738, *P* < 0.05], followed by Tukey’s multiple comparison test. Moreover, these rats also exhibited significantly higher values of GluA1ps831 compared to all other groups (*P* < 0.05), as determined by a two-way ANOVA test. [*F*_OPTIC STIMULATION_ (1,10) = 6.500, *P* < 0.05, *F*_GROUP_ (1,10) = 5.396, *P* < 0.05, *F*_INTERACTION_ (1,10) = 4.019, *P* > 0.05], followed by Tukey’s multiple comparison test. Furthermore, enhancing the GABAergic tone originating from the RMTg during cocaine conditioning attenuates changes in pERK, GluA1ps845, and GluA1ps831 in the NAc (Fig. 3B, D, E). It is important to note that in some of the experiments done in the NAc the number of samples was relatively low (*N* = 3) due to technical issues of tissue processing and therefore the interpretation of the results should be taken with care.

**Fig. 4:**
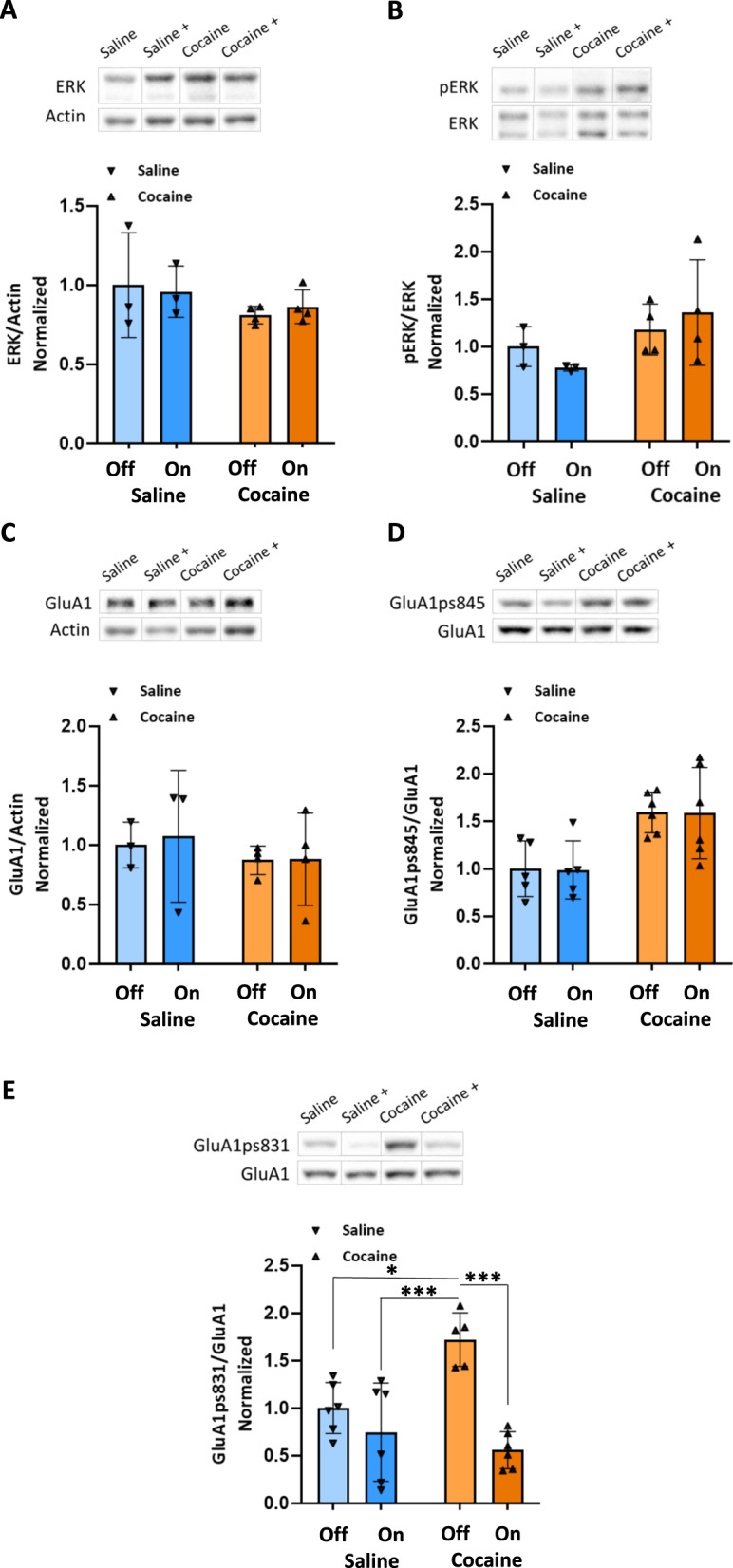
Enhanced GABAergic tone from the RMTg during conditioning to cocaine attenuates changes in GluA1ps831 but not GluA1ps845 in the PFC. All rats were euthanized 45 min after the RePP test. Then, their PFCs were taken out and homogenized. Samples were resolved by SDS-PAGE, and WB membranes were probed with the appropriate antibodies. **A** The bar histograms depict the level of ERK divided by Actin, no significant difference was found (*P* > 0.05), as determined by a two-way ANOVA test [*F*_OPTIC STIMULATION_ (1,10) = 0.003651, *P* > 0.05, *F*_GROUP_ (1,10) = 2.211, *P* > 0.05, *F*_INTERACTION_ (1,10) = 0.2461, *P* > 0.05, followed by Tukey’s multiple comparison test] (*n* = 3 for the saline groups, *n* = 4 for the cocaine groups). **B** The bar histograms depict the level of pERK (Thr202/Tyr204) divided by ERK protein, no significant difference was found (*P* > 0.05), as determined by a two-way ANOVA test [*F*_OPTIC STIMULATION_ (1,10) = 0.01467, *P* > 0.05, *F*_GROUP_ (1,10) = 4.050, *P* > 0.05, *F*_INTERACTION_ (1,10) = 1.125, *P* > 0.05, followed by Tukey’s multiple comparison test]. (*n* = 3 for the saline groups, *n* = 4 for the cocaine groups). **C** The bar histograms depict the level of GluA1 divided by Actin, no significant difference was found (*P* > 0.05), as determined by a two-way ANOVA test [*F*_OPTIC STIMULATION_ (1,10) = 0.04842, *P* > 0.05, *F*_GROUP_ (1,10) = 0.7459, *P* > 0.05, *F*_INTERACTION_ (1,10) = 0.03048, *P* > 0.05, followed by Tukey’s multiple comparison test]. (*n* = 3 for the saline groups, *n* = 4 for the cocaine groups). **D** The bar histograms depict the level of GluA1ps845 divided by GluA1 protein, rats treated with cocaine (15 mg/kg) displayed significantly higher values compared to the saline without optic stimulation and saline with optic stimulation groups (*P* < 0.05), no significant difference was found between the cocaine group and cocaine with optic stimulation group (*P* > 0.05), as determined by a two-way ANOVA test [*F*_OPTIC STIMULATION_ (1,18) = 0.003881, *P* > 0.05, *F*_GROUP_ (1,18) = 16.48, *P* < 0.001, *F*_INTERACTION_ (1,18) = 0.0005230, *P* > 0.05, followed by Tukey’s multiple comparison test]. (*n* = 5 for the saline groups, *n* = 6 for the cocaine groups). **E** The bar histograms depict the level of GluA1ps831 divided by GluA1 protein, rats treated with cocaine (15 mg/kg) displayed significantly higher values compared to all other groups (*P* < 0.05 for saline- optic stimulation, *P* < 0.001 for saline + optic stimulation and cocaine+ optic stimulation groups), as determined by a two-way ANOVA test [*F*_OPTIC STIMULATION_ (1,19) = 24.75, *P* < 0.0001, *F*_GROUP_ (1,19) = 3.460, *P* > 0.05, *F*_INTERACTION_ (1,19) = 10.23, *P* < 0.01, followed by Tukey’s multiple comparison test]. (*n* = 6 for the saline groups, *n* = 5 and 6 for the cocaine– and cocaine+ groups, respectively). (Off refers to –optic stimulation, whereas On refers to +optic stimulation).

Finally, in the PFC (Fig. 4), rats treated with cocaine displayed significantly higher values of GluA1ps845 compared to the saline without optic stimulation and saline with optic stimulation groups (*P* < 0.05), as determined by a two-way ANOVA test [*F*_OPTIC STIMULATION_ (1,18) = 0.003881, *P* > 0.05, *F*_GROUP_ (1,18) = 16.48, *P* < 0.001, *F*_INTERACTION_ (1,18) = 0.0005230, *P* > 0.05], followed by Tukey’s multiple comparison test. Additionally, these rats displayed significantly higher values of GluA1ps831 compared to all other groups (*P* < 0.05 for saline – optic stimulation, *P* < 0.001 for saline + optic stimulation and cocaine + optic stimulation groups), as determined by a two-way ANOVA test [*F*_OPTIC STIMULATION_ (1,19) = 24.75, *P* < 0.0001, *F*_GROUP_ (1,19) = 3.460, *P* > 0.05, *F*_INTERACTION_ (1,19) = 10.23, *P* < 0.01], followed by Tukey’s multiple comparison test. Furthermore, enhancing the GABAergic tone originating from the RMTg during cocaine conditioning attenuates changes in GluA1ps831 but not GluA1ps845 (Fig. 4E, D). Importantly, optic stimulation of RMTg afferents paired with saline exposure during conditioning does not affect GluA1 and ERK in the VTA, NAc, and PFC compared to the group that received saline without optic stimulation (Figs. 2, 3, and 4).

In summary, the results suggest that when the GABAergic transmission from the RMTg is enhanced during the conditioning process with cocaine, it leads to a reduction in the molecular changes that typically occur in VTA, NAc, and PFC as a result of cocaine exposure. This modulation of GABAergic transmission is also associated with a decreased preference for the chamber that is associated with cocaine in a CPP test, referred to as the RePP test.

## Discussion

In this study, our aim was to investigate the effects of activating the inputs from the RMTg to the VTA on cocaine-induced molecular changes in the VTA, NAc, and PFC. To accomplish this, we conducted a shortened CPP experiment to accelerate the development of conditioned preference. To assess whether the optic stimulation alone could influence the behavioral outcomes of CPP and the molecular profile in the brain regions of interest we included two additional control groups. The first group received saline without optic stimulation, while the second group received saline with optic stimulation. Using optogenetics, we selectively activated GABAergic inputs from the RMTg to the VTA during cocaine exposure in real-time throughout the conditioning period. This stimulation was applied during the initial 15 min following cocaine injections, aligning with the opponent process theory that suggests a shift from pleasurable to aversive effects after this period [41]. Similar to the longer CPP presented previously [37], we found that activating the RMTg-VTA GABAergic inputs during cocaine conditioning significantly reduced the preference for the cocaine-associated chamber in the RePP test. Moreover, optic stimulation of RMTg-VTA afferents reverses cocaine-induced molecular changes in the expression and phosphorylation state of ERK and GluA1. Our results provide additional support for the regulatory role of the RMTg in the reward system, particularly in the context of psychostimulant effects. Further research in this area has the potential to lead to the development of new interventions or treatments for individuals facing challenges with cocaine addiction.

The observed elimination of preference for the cocaine-associated chamber in animals undergoing RMTg stimulation during cocaine conditioning suggests that the activation of RMTg afferents interferes with the rewarding effects of cocaine, potentially by attenuating cocaine-induced neuroadaptations in the brain’s reward circuitry. These neuroadaptations are not restricted to the VTA but also occur in brain regions that receive input from DA neurons originating in the VTA, including the NAc and the PFC. In the VTA, cocaine increases DA levels by blocking DAT. DA activates D1 receptors (D1R) on VTA neurons, leading to downstream signaling through protein kinase A (PKA). PKA directly phosphorylates the GluA1 subunit of AMPA receptors at S845 [42] and activates ERK signaling, resulting in increased pERK levels [43]. Activation of RMTg-VTA inputs reduces neuronal activity in VTA DA neurons [37] by activating GABA_A_ receptors (GABA_A_R). This decreases DA secretion and D1R activation, leading to reduced PKA activation, pERK levels, and GluA1 phosphorylation at S845 [42, 44]. Additionally, GABA_A_R activation reduces intracellular calcium (Ca^2+^) levels [45], contributing to a decrease in GluA1 phosphorylation at S831. Dephosphorylation of GluA1 at S831 is involved in long-term depression (LTD) induction in previously potentiated synapses and the decrease in cell surface AMPA receptors during LTD [46, 47].

In the NAc, cocaine increases pERK, GluA1ps845, and GluA1ps831 levels. These changes may contribute to alterations in synaptic plasticity and reward-related processes within this brain region. Previous studies have shown that acute cocaine administration does not alter glutamate release in the accumbens of naïve animals but produces marked glutamate release in animals previously treated with repeated cocaine, especially when cocaine is associated with environmental cues [48, 49]. The enhanced release of glutamate occurs against a background of significantly reduced basal levels of glutamate in the extracellular space and inside presynaptic terminals [50, 51]. This reduction in the glutamate tone may amplify the synaptic signal transmitted by the released glutamate from the PFC to the NAc [49, 50]. Glutamate, acting on NMDA receptors (NMDAR), triggers calcium (Ca^2+^) influx, leading to the activation of Ca^2+^/ Calmodulin-Dependent Protein Kinase II (CaMKII). CaMKII phosphorylates the GluA1 subunit of AMPA receptors at S831 [42]. During cocaine withdrawal, there is a known increase in the cAMP-PKA signaling pathway in the NAc [52]. The upregulation involves increased activity of adenylyl cyclase and PKA due to repeated cocaine administration. However, these increases in adenylyl cyclase and PKA activity diminish within the first three days after discontinuing cocaine injections. During early withdrawal, the upregulation of PKA signaling may facilitate the regulation of AMPAR trafficking in the NAc. PKA activation phosphorylates the GluA1 subunit of AMPA receptors at S845 [42]. In addition, PKA activation is associated with the activation of ERK signaling pathways, leading to increased pERK levels.

Activation of RMTg-VTA inputs attenuates the changes observed in pERK, GluA1ps845, and GluA1ps831. The decrease in pERK and GluA1ps845 levels is likely due to reduced DA secretion from the VTA, leading to reduced activation of D1R in the NAc. The reduction in the GluA1ps831 levels is probably caused by decreased glutamate release from the PFC to the NAc. This decreased glutamate release results in reduced activation of NMDA receptors (NMDARs) and, consequently, decreased calcium (Ca^2+^) influx into the cell [45]. As a result, decreasing the activity of CaMKII and reduced phosphorylation of the GluA1 subunit of AMPA receptors at S831.

In the PFC, we observed an increase in the levels of GluA1ps845 and GluA1ps831, indicating alterations in the phosphorylation state of the GluA1 subunit of AMPA receptors. The membrane potential of pyramidal cells in the PFC normally fluctuates between relatively depolarized and hyperpolarized potentials, regulated by dopaminergic and glutamatergic inputs. This fluctuation reflects the tonic activity in the cortical circuitry [53]. Repeated cocaine exposure disrupts the normal fluctuation and leads to the loss of membrane bistability in the PFC. This loss could be due to changes in the pyramidal cells themselves or changes in dopaminergic and/or glutamatergic inputs to the PFC. Previous studies have shown that NMDA receptor-dependent long-term potentiation (LTP) can be induced in the PFC through tetanic stimulation of hippocampal projections. Furthermore, this LTP can be enhanced by DA or VTA stimulation and reduced by VTA lesions [54]. Additional studies have demonstrated that activation of D1 receptors (D1R), but not D2 receptors (D2R), prior to tetanus stimulation facilitates LTP through a PKA-dependent mechanism [55, 56]. D1R activation stimulates the cAMP-PKA signaling pathway, leading to the activation of PKA. PKA directly phosphorylates the GluA1 subunit of AMPA receptors at S845 [42]. In addition, PKA activation accompanied by NMDAR activation by glutamate activates voltage-gated calcium channels (VGCCs). Calcium influx into the postsynaptic neuron triggers the formation of a calcium-calmodulin complex, which activates CaMKII. CaMKII directly phosphorylates the GluA1 subunit of AMPA receptors at S831 [42]. These phosphorylation events of GluA1 at S845 and S831 contribute to synaptic plasticity processes, including the expression of LTP.

Activation of RMTg-VTA inputs attenuates changes in GluA1ps831 in the PFC. Studies have demonstrated that LTD induction in previously potentiated synapses is associated with dephosphorylation of S831 [46, 47]. The reduction in GluA1ps831 levels is likely caused by decreased glutamate release, possibly from hippocampal projections to the PFC, which leads to less activation of NMDA receptors, resulting in decreased CaMKII activity and reduced phosphorylation of GluA1 at S831. The involvement of the NAc and PFC suggests that these neuroadaptations extend beyond the initial site of DA release and affect downstream regions involved in reward processing and executive functions. These neuroadaptations in the NAc and PFC may contribute to reinforcing the rewarding effects of cocaine and the development of addictive behaviors. Importantly, the comparison between the saline + optic stimulation group and the saline–optic stimulation group did not reveal any significant differences in the VTA, NAc, and PFC. This suggests that the activation of RMTg-VTA inputs alone, without the influence of cocaine, does not lead to observable changes in these brain regions associated with reward processing. This indicates that the activation of RMTg-VTA inputs specifically affects neuroadaptations induced by cocaine, rather than having a general effect on reward-related areas. It is important to note that in the current study, rats were sacrificed for 45 min following the behavioral procedures (test), and the phosphorylation state of GluA1 and ERK were tested right after. Phosphorylation events usually return to the baseline state 10–20 min following their stimulation. However, we have previously shown that phosphorylation of ERK after repeated cocaine exposure remained elevated one or even 3 weeks following withdrawal [39], although long-lasting changes in ERK phosphorylation can be a distinct process from acute ERK phosphorylation. Together, our results suggest that these events highly depend on the mode of activation.

A popular current hypothesis is that cocaine addiction is due to drug-induced adaptations in the cellular mechanisms that underlie normal learning and memory. Such mechanisms involve signaling mediated by ERK which is critical for cocaine reward (as measured by CPP) and consolidation and reconsolidation of memories for cocaine cue. In addition, it also involves changes in AMPAR signaling including an increase in AMPAR expression and function, changes in AMPAR trafficking, and altered phosphorylation and subunit composition [39]. In the current study, we demonstrated that optical stimulation of the RMTg-VTA pathway attenuated cocaine-induced biochemical changes in addiction-related brain regions, with a specific focus on the VTA, NAc, and PFC. However, we can not rule out the possibility that this manipulation has the potential to influence other brain regions related to learning that were not addressed in our study, thus potentially impacting other learning tasks. However, it’s worth emphasizing that this manipulation was combined with cocaine injections to specifically target cocaine reward and, consequently, to affect the learning processes associated with exposure to cocaine.

Our current findings highlight the importance of this circuitry in the context of cocaine addiction and suggest that it could be a target for therapeutic interventions aimed at mitigating the effects of cocaine on the brain. It’s worth noting that the interpretation of the study’s findings should be based on the specific research context and methodology. The understanding of these complex neural processes is continually evolving, and additional research is needed to further explore the mechanisms and implications of RMTg-VTA circuitry in relation to cocaine addiction and reward processing.

## Data Availability

The dataset generated and analyzed during the current study is available from the corresponding author upon reasonable request.
